# Effects of colchicine on depression scale scores of patients with chronic heart failure with reduced ejection fraction: A randomized clinical trial

**DOI:** 10.34172/jcvtr.025.33169

**Published:** 2025-09-28

**Authors:** Mohammadreza Taban-Sadeghi, Sajad Khiali, Mahsa Karimi, Mehrdad Shahidi, Elnaz Khani, Zahra Mousavi, Taher Entezari-Maleki

**Affiliations:** ^1^Cardiovascular Research Center, Tabriz University of Medical Sciences, Tabriz, Iran; ^2^Department of Clinical Pharmacy, Faculty of Pharmacy, Tabriz University of Medical Sciences, Tabriz, Iran; ^3^Department of Psychiatry, Tabriz University of Medical Sciences, Tabriz, Iran

**Keywords:** Clinical trial, Heart failure, HFrEF, Depression, Colchicine

## Abstract

**Introduction:**

Since there is a bidirectional interaction between heart failure (HF) and depression, we aimed to evaluate the effects of colchicine on depression scale scores in individuals with HF with reduced ejection fraction (HFrEF).

**Methods:**

A randomized clinical trial was carried out on 50 patients with HFrEF. The intervention group (n=25) received colchicine 0.5 mg twice a day plus standard care for HFrEF, while the control group (n=25) received only standard care. The study’s primary outcome was the changes in the 21-item Hamilton Depression Rating Scale (HDRS-21) over 12 weeks.

**Results:**

During the study period, HDRS-21 significantly decreased in both groups (both *P*<0.05); however, there were no significant differences between the study groups at baseline (12.2±2.9 vs. 10.5±2.9; *P*=0.54), week 4 (6.6±2 vs. 5.8±4.7; *P*=0.21), and week 12 (2.1±2 vs. 4.2±4.5; *P*=0.11).

**Conclusion:**

This study shows that colchicine had no significant effect on HDRS-21 in individuals with HFrEF. Further studies are warranted to confirm the study hypothesis.

## Introduction

 Cardiovascular disease (CVD) is the leading cause of mortality worldwide, according to the American Heart Association (AHA) statistics.^1^ The prevalence of heart failure (HF) is increasing due to population aging and the improvement in managing patients with ischemic heart disease (IHD). It has been estimated that HF with reduced ejection fraction (HFrEF) accounts for about 50% of all HF cases. Despite progress in treating patients with HFrEF, its morbidity and mortality remain high.^2^

 Depression is common in patients with HF and is associated with several physiological derangements that could contribute to adverse outcomes such as reduced adherence to treatment, progression of HF, an increased hospitalization rate, and mortality.^3,4^

 It has been proposed that distribution in the peripheral immune system and subsequent over-activation of pro-inflammatory cytokines, including interleukin 1 (IL-1), IL-6, and tumor necrosis factor-alpha (TNF-α), have long been associated with the development of mood disorders. Disturbances in leukocyte function and/or leukocyte number and elevated cytokine expression have been proposed to play a fundamental role in the development of depression.^5^ There is also increasing evidence that anti-inflammatory medications may have antidepressant effects in patients with depression.^6^

 The United States (U.S.) Food and Drug Administration (FDA) recently approved colchicine as the first anti-inflammatory medication for reducing cardiovascular event risks, making a significant milestone in the emerging field of CVD inflammation treatment.^7^ Colchicine could inhibit NOD-like receptor protein 3 (NLRP3) inflammasome that contributes to caspase-1 activation and subsequent IL1β and IL18 release.^8^ It was also shown that colchicine decreases lipopolysaccharide-related TNFα secretion by macrophages in a rat model.^9^ In a meta-analysis of 8 studies including 6016 patients with acute and chronic coronary syndromes, colchicine was associated with a reduction in hs-CRP, which has a role in depressive symptoms.^10,11^ It also decreases the expression of TNF receptors on macrophages and endothelial cells by increasing the level of cyclic adenosine monophosphate.^12,13^

 Although no evidence directly assesses the anti-depressant effects of colchicine, it has been proposed that colchicine may decrease depression in bipolar patients by mitigating the pro-inflammatory effects associated with lithium treatment.^14^

 Given the above, we aimed to evaluate the effects of colchicine on the 21-item Hamilton Depression Rating Scale (HDRS-21) in individuals with HFrEF.

## Materials and Methods

###  Ethics

 The study was reviewed and approved by all relevant institutional review boards and regulatory authorities. The study protocol was approved by the Research Ethics Committee of the Tabriz University of Medical Sciences (the ethics number is IR.TBZMED.REC.1401.449) and then was verified in the International Clinical Trials Registry Platform with identification IRCT20111206008307N43 (date of first trial registration: July 12, 2022). The present study was carried out in accordance with the Declaration of Helsinki and later revisions on ethical principles for medical research.^15^ All patients were informed about how the study would be conducted. Written informed consent for participation was obtained from patients or their guardians. Patients were free to withdraw from the study at any time without giving any reason, and their medical care and legal rights would not be affected. The patients’ information will remain confidential to the researchers.

###  Study design and setting

 This pilot study is a prospective, parallel-group, randomized clinical trial conducted in the Shahid Madani Heart Center (SMHC), the largest teaching referral hospital for CVDs affiliated with Tabriz University of Medical Science, from December 2022 to June 2023.

###  Study population 

 All consented patients aged between 18 and 80 years with a confirmed diagnosis of HFrEF entered the study. Patients with estimated glomerular filtration rate (eGFR) less than 30 mL/min/1.73 using the Chronic Kidney Disease Epidemiology Collaboration (CKD-EPI) formula, liver failure with Child-Pugh score of equal or more than 7 (Grade B or C), inflammatory and autoimmune disease, moderate or severe depression based on the HDRS-21 to maintain homogeneity of the study population, current or prior use of anti-depressant medications with 4 weeks, chronic diarrhea, history of cancer within the previous three years, current or planned long-term systemic glucocorticoid therapy, neuromuscular disease, clinically significant non-transient blood dyscrasia, drug or alcohol abuse, females of childbearing potential without adequate contraceptive methods, pregnancy, lactation, and contraindications of colchicine or other medications were excluded from the study.

###  Randomization and study process

 Patients were assigned to the intervention (n = 25) and the control (n = 25) groups using the systematic randomization method (computer-generated random numbers by online Graphpad prism randomization) by an independent person who was not involved in the study process. All researchers who were involved directly in the study process and patients were blind throughout the study period and were unaware of the treatment administered.

 In the intervention group, patients received colchicine 0.5 mg twice daily orally for 12 weeks. All of the drugs used in this study are generic, with no specific brand. All patients were treated based on the 2022 AHA, the American College of Cardiology (ACC), and the Heart Failure Society of America (HFSA) guidelines for the management of HFrEF. Baseline demographic and clinical data of patients, such as sex, age, drug history, past medical history, laboratory data, and positive family history of CVD, were recorded in data collection forms. A blinded psychiatrist assessed the depression of patients by the HDRS-21 (a widely used tool for assessing the severity of depressive symptoms in clinical trials)^16,17^ at baseline, week 4, and week 12. The patients’ compliance with the use of medications was assessed using the pill counting method with above 80 % use of their medicines in each course of the visit.

###  Study Endpoints

 The primary endpoint of the study was the change in HDRS-21 in baseline, weeks 4 and 12. The occurrence of adverse events was also assessed.

###  Statistical analysis

 Data analysis was carried out in IBM SPSS Statistics for Windows 16 (SPSS Inc., Chicago, Illinois, 2007). First, to check the normality of the data distribution, the Kolmogorov-Smirnov test was used. Data were shown as number (%) for categorical variables, mean ± standard deviation (SD) for normal continuous variables, and median (interquartile range (IQR)) for non-normal continuous variables. For between-group analyses, the independent t-test or Mann–Whitney U test was applied to categorical variables, as appropriate. The chi-square or Fisher’s exact test was used for categorical variables. For within-group analysis, repeated measure analysis of variance (ANOVA) or Friedman test was applied. The *P* value under 0.05 was noted to be statistically significant.

###  Sample size calculation

 Due to the pilot nature of the study, we selected a sample size of about 50 patients as a routine of most pilot studies. We also calculated the post-hoc power of the study using the effect size of the primary outcome to show that the selected sample size is adequate. The power of the study was estimated using GPower version 3.1. Assuming the sample size of 50, three times measurements, 95% confidence interval, and α = 0.05, the estimated power for change in HDRS-21 with partial η2 of 0.21 and effect size of 0.51 was 100%, demonstrating the sufficiency of the sample size.

## Results

###  Study patients 

**Figure 1:**
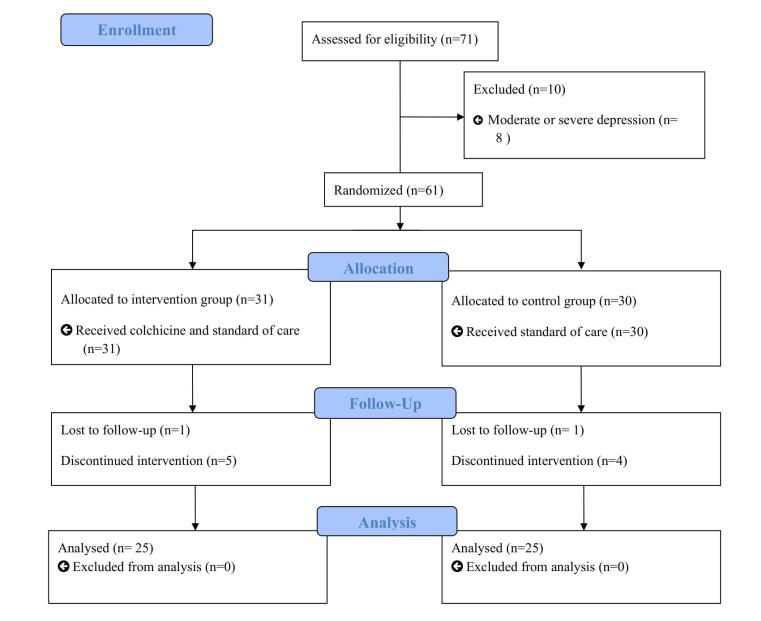
CONSORT flow diagram of the study

 A total of 71 patients were evaluated for eligibility. Among them, 10 patients were not included due to moderate to severe depression and were referred to the psychiatrist (n = 8) and kidney failure with eGFR under 30 mL/min/1.73 (n = 2). So, 61 patients were included in the study and randomized to the intervention (n = 31) and control (n = 30) groups. Finally, 50 patients (25 in each group) completed the trial. The missing data is unrelated to the study outcomes (Figure 1). Our study population has a low socio-economic status. Most patients were male (72% in the intervention and 80% in the control groups). The participants’ ages were 63.5 ± 8 in the intervention group and 61.08 ± 7.5 in the control group (*P* = 0.570). The most commonly used medications were angiotensin-converting enzyme inhibitor (ACEI), angiotensin receptor blocker (ARB), angiotensin receptor–neprilysin inhibitor (ARNI), sodium-glucose cotransporter-2 (SGLT2) inhibitor, and beta-blocker. As shown in Table 1, there was no significant difference regarding the baseline demographic and clinical data of patients between the study groups (*P* > 0.05).

**Table 1:** Demographic and clinical data of the study patients

**Demographic/Clinical Data**	**Intervention (n=25)**	**Control (n=25)**	***P*** **-value**
Age (years), mean ± SD	63.5 ± 8	61.1 ± 7.5	0.570
Sex, male, n (%)	18 (72)	20 (80)	0.508
Body mass index (kg/m^2^), mean ± SD	28.7 ± 4.3	27.6 ± 5.2	0.538
Serum creatinine (mg/dl), mean ± SD	1.5 ± 0.4	1.5 ± 0.5	0.612
eGFR (ml/min/1.73m), mean ± SD	62.2 ± 28.6	68.2 ± 28.5	0.520
Fasting blood glucose (mg/dl), mean ± SD	142.3 ± 62.5	138 ± 83	0.731
Left ventricular ejection fraction (%), mean ± SD	24.9 ± 10.5	25 ± 10.8	0.880
Hypertension, n (%)	7 (28)	8 (32)	0.512
Family history of CVDs, n (%)	8 (32)	12 (48)	0.737
ACEIs/ARBs, n (%)	21 (84)	20 (80)	0.567
Angiotensin receptor/neprilysin inhibitors, n (%)	2 (8)	2 (8)	> 0.99
Calcium channel blockers, n (%)	2 (8)	2 (8)	> 0.99
Beta blockers, n (%)	19 (76)	21 (84)	0.768
Antihyperlipidemic drugs, n (%)	19 (76)	18 (72)	0.654
Sodium-glucose cotransporter-2 inhibitors, n (%)	25 (100)	25 (100)	> 0.99
Diuretics, n (%)	11 (44)	14 (56)	0.778
Proton pump inhibitors, n (%)	7 (28)	8 (32)	0.860
Mineralocorticoid receptor antagonist, n (%)	9 (36)	13 (52)	0.658

SD, standard deviation; ACEI, Angiotensin-converting enzyme inhibitor; ARB, Angiotensin II receptor blocker; CVD, cardiovascular disease; eGFR, estimated glomerular filtration rate

###  Study outcomes

**Figure 2:**
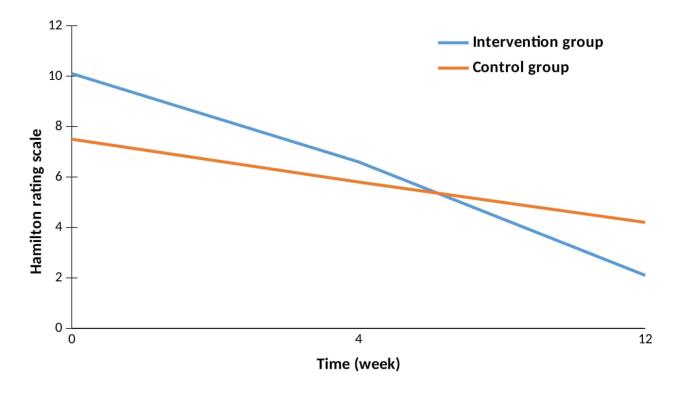
The changes in the Hamilton Depression Rating Scale during the study period

 Baseline HDRS-21 of the patients was comparable between groups the intervention (10.1 ± 4) and control (7.5 ± 3.7) groups regarding (*P* = 0.054). During the study period, the HDRS-21 significantly decreased in both groups (intervention,* P* = 0.002; control, *P* = 0.001); however, no significant difference was observed regarding HDRS-21 in week 4 and week 12 between the intervention and control groups, respectively (week 4: 6.6 ± 2.1 vs. 5.8 ± 4.7, *P* = 0.216; week 12: 2.1 ± 2 vs. 4.2 ± 4.5; *P* = 0.109) (Table 2) (Figure 2). In terms of safety, colchicine was well-tolerated, and no serious adverse event leading to drug discontinuation WAS observed during the study follow-up period.

**Table 2:** Mean Hamilton Depression Rating Scale at baseline, month 1 and month 3 in the study groups

**Hamilton scale**	**Intervention (n=25)**	**Control (n=25)**	***P*** **-value**
Baseline	10.1 ± 4	7.5 ± 3.7	0.054
Month 1	6.6 ± 2.1	5.8 ± 4.7	0.216
Month 3	2.1 ± 2	4.2 ± 4.5	0.109

## Discussion

 To our knowledge, this is the first randomized clinical trial to assess the effect of colchicine on HDRS in patients with HFrEF. The results of this study showed that adding colchicine 0.5 mg twice daily to the guideline-directed medical therapy (GDMT) based on the 2022 AHA/ACC/HFSA guideline for the management of HFrEF had no significant effect on patients’ depression score during the study period of 12 weeks.

 Colchicine exerts anti-inflammatory features in cardiovascular disease, particularly in the context of atherosclerosis. Its mechanisms of action include inhibiting inflammatory pathways, modulation of immune cell activity, and stabilization of atherosclerotic plaques.^18^ Colchicine inhibits the NLRP3 inflammasome, decreasing the release of inflammatory cytokines such as IL-1β and IL-18, which have deleterious effects on cardiovascular diseases.^8^ Colchicine also modulates immune cell activity by reducing neutrophil recruitment and the formation of Neutrophil Extracellular Traps (NETs), which are important in inflammation exacerbation and thrombosis.^18^ Furthermore, it enhances the structural integrity of plaques and reduces the risk of rupture by promoting the deposition of collagen in plaques.^18^

###  Depression and HF

 Based on the literature, there is a well-established correlation between depression and HF. The prevalence of depressive mood disorders is common in individuals with HF. A systematic review and meta-analysis of 149 studies, including 305,407 individuals with HF, showed that the global prevalence of any severity and moderate to severe severity of depression was 41.9% and 28.1%, respectively.^19^

 Furthermore, depression dramatically worsens the quality of life and clinical outcomes and increases the risk of HF, particularly in high-risk individuals.^20^ It has been HF individuals with depression have a 2-fold risk of emergency room visits and markedly higher hospital readmission rates compared with those HF without depression.^21^ A systematic review and meta-analysis of nine studies, including 4012 patients with HF, showed that the pooled hazard ratio of all-cause mortality was 1.51 (95% CI 1.19–1.91) for depression compared with individuals without depression.^22^

 Although the pathophysiology of both HF and depression is poorly understood, several pathways and risk factors are common to both illnesses. These include platelet dysregulation, inflammation, and neuroendocrine dysfunction.^4^ Depression could also worsen the clinical outcomes of individuals with HF through non-adherence to treatment and decreased performance.^23^ Given the bilateral correlation between HF and depression, both ACC/AHA and ESC HF guidelines advise increasing the awareness of depressive disorders in patients with HF and appropriate management.^24,25^

###  Colchicine in HF

 Colchicine has long been used as a safe and well-tolerated treatment for gout and familial Mediterranean fever. However, the widely accessible treatment includes various anti-inflammatory properties that have been shown to be useful in a wide range of cardiovascular disorders.^12^ Low-dose colchicine was recently approved by the U.S. FDA as the first anti-inflammatory atheroprotective cardiovascular treatment to reduce the risk of myocardial infarction (MI), stroke, coronary revascularization, and cardiovascular death in patients with established atherosclerotic disease or multiple risk factors for CVD.^7^ However, the effects of colchicine in the management of HF remain a controversial issue.

 A growing body of evidence supports the crucial role of inflammation in the pathophysiology of HF. It has been indicated that levels of circulating inflammatory markers, such as C-reactive protein (CRP) and IL-6, are elevated in patients with HF and could worsen cardiac dysfunction.^26,27^ It has been hypothesized that colchicine could improve oxidative stress, cardiac function, and clinical outcomes of patients with HF through inhibition of the nucleotide-binding domain, leucine-rich–containing family, pyrin domain–containing-3 inflammasome, which is a multiprotein complex key role in the activation of the innate immune system and the production of pro-inflammatory cytokines.^28^

 Deftereos et al in a double-blinded, placebo-controlled trial in 267 individuals with stable symptomatic HF and EF ≤ 40%, showed that adding colchicine 0.5 mg twice daily to standard care had no significant effects on the composite of mortality or hospitalization duration for HF (9.4% vs. 10.1%; *P* = 0.839) as well as the performance of patients (*P* = 0.938). However, inflammatory biomarkers CRP and IL-6 were significantly reduced in the colchicine group, compared with the placebo (both *P* < 0.001).^29^ In our study, adding colchicine 0.5 mg twice to standard care for 12 weeks did not significantly decrease the HDRS of patients with HF. This could be due to a partially small sample size and the short follow-up duration of the study.

## Strengths and limitations of the study

 The present study evaluated the efficacy and safety of colchicine on depression for the first time in patients with HFrEF. The study was double-blinded and randomized and depressive symptoms were assessed by only one researcher and a single method that decreased the risk of selection and measurement bias. First, due to the pilot nature of the study, we performed this study in one center with no placebo. Second, due to time and cost constraints, the duration of the study was short, and the sample size was relatively small, which may be potential reasons for the lack of significant findings. Third, to minimize the confounding effects of antidepressant medications, patients with moderate or severe depression were excluded from the trial, which could limit the generalizability of the findings to those with moderate to severe depression. Although we did not perform an analysis of the intention to treat, the missing data in this research is unrelated to the study outcomes, which may not raise bias arising from missing data. Finally, we did not assess lifestyle factors, such as education level, diet, and physical activity, in our study.

## Conclusion

 According to the results obtained from the study, the use of 0.5 mg of colchicine twice a day along with the standard treatments for HFrEF in the intervention group didn’t have a significant effect on reducing the HDRS-21 in patients with HFrEF. Further well-designed studies with larger sample sizes and longer duration that use alternative dosages of colchicine are required to confirm this conclusion.

## Competing Interests

 The authors declare that they have no competing interests.

## Ethical Approval

 The trial was authorized by the research ethics committee of the Tabriz University of Medical Sciences (Ethics number: IR.TBZMED.REC.1401.370), and registered in the International Clinical Trials Registry Platform with the identifier IRCT20111206008307N39.
